# Ocular toxicity of authentic lunar dust

**DOI:** 10.1186/1471-2415-12-26

**Published:** 2012-07-20

**Authors:** Valerie E Meyers, Hector D Garcìa, Kathryn Monds, Bonnie L Cooper, John T James

**Affiliations:** 1 Space Toxicology Office, NASA Johnson Space Center, 2101 NASA Parkway, MC: SF23, Houston, 77058, TX; 2 Space Toxicology Office, Wyle Science, Technology and Engineering Group, 1290 Hercules Drive, Houston, 77058, TX; 3 Stillmeadow, Inc., 12852 Park One Drive, Sugar Land, 77478, TX; 4 Jacobs Technology, 2224 Bay Area Boulevard, Houston, 77058, TX

## Abstract

**Background:**

Dust exposure is a well-known occupational hazard for terrestrial workers and astronauts alike and will continue to be a concern as humankind pursues exploration and habitation of objects beyond Earth. Humankind’s limited exploration experience with the Apollo Program indicates that exposure to dust will be unavoidable. Therefore, NASA must assess potential toxicity and recommend appropriate mitigation measures to ensure that explorers are adequately protected. Visual acuity is critical during exploration activities and operations aboard spacecraft. Therefore, the present research was performed to ascertain the ocular toxicity of authentic lunar dust.

**Methods:**

Small (mean particle diameter = 2.9 ± 1.0 μm), reactive lunar dust particles were produced by grinding bulk dust under ultrapure nitrogen conditions. Chemical reactivity and cytotoxicity testing were performed using the commercially available EpiOcular^TM^ assay. Subsequent *in vivo* Draize testing utilized a larger size fraction of unground lunar dust that is more relevant to ocular exposures (particles <120 μm; median particle diameter = 50.9 ± 19.8 μm).

**Results:**

*In vitro* testing indicated minimal irritancy potential based on the time required to reduce cell viability by 50% (ET50). Follow-up testing using the Draize standard protocol confirmed that the lunar dust was minimally irritating. Minor irritation of the upper eyelids was noted at the 1-hour observation point, but these effects resolved within 24 hours. In addition, no corneal scratching was observed using fluorescein stain.

**Conclusions:**

Low-titanium mare lunar dust is minimally irritating to the eyes and is considered a nuisance dust for ocular exposure. No special precautions are recommended to protect against ocular exposures, but fully shielded goggles may be used if dust becomes a nuisance.

## Background

**Figure 1:**
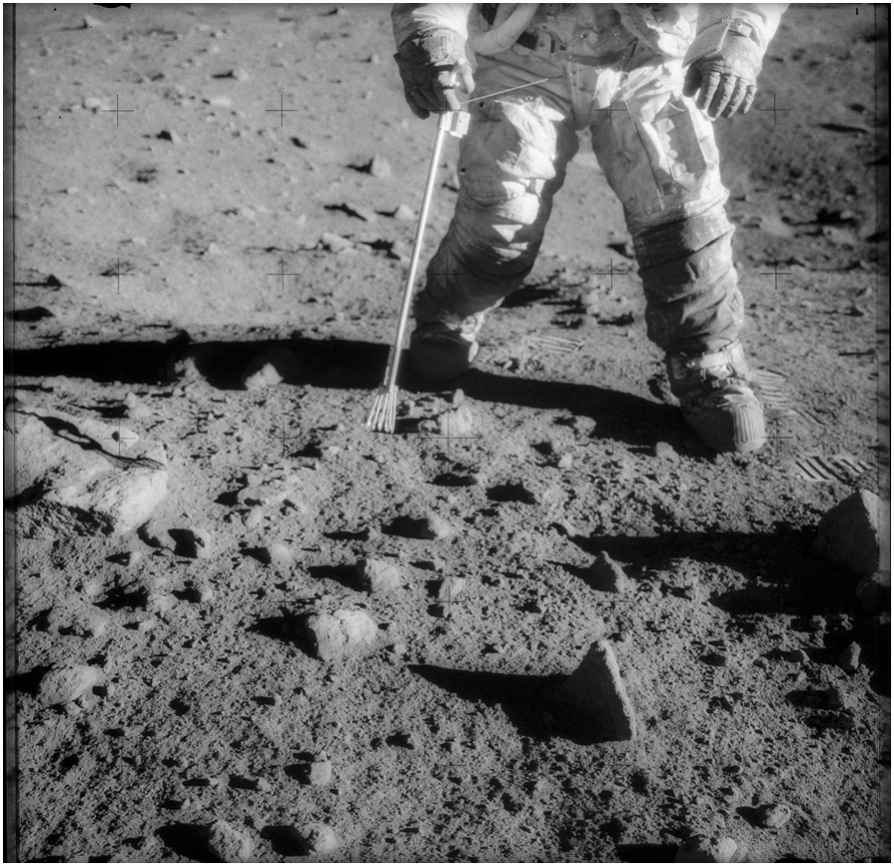
**Apollo 12 spacesuit.** An image depicting the lunar surface and dust collecting on the spacesuit of an Apollo astronaut.

According to the Centers for Disease Control and Prevention, about 2000 US workers have medically relevant job-related eye injuries each day [1]. The majority of these injuries result from small particles, including dust, impacting or abrading the eye. The lunar regolith, which includes dust, is a product of billions of years of meteorite impacts, micrometeorite impacts, cosmic dust, solar wind hydrogen implantation, and ionizing radiation. It is several meters thick in all areas where it has been measured [2]. Current experience with human exposure to lunar dust is limited to the Apollo program. Astronauts who explored the lunar surface acquired large amounts of dust on their spacesuits (Figure 1), which returned with them in their spacecraft. When the vehicle left the lunar surface and returned to microgravity operations on the return trip to Earth, the lunar dust became airborne and was reported to be irritating to the eyes of Apollo astronauts [3]. The crew’s response at the time was to simply don their helmets until the dust was cleared from the atmosphere of the vehicle by filters in the environmental control and life support system. No reports of injury were found in the available NASA records.

NASA anticipates that long stays on the surface of the Moon or other celestial bodies will provide many opportunities to bring surface dust back into the living areas, both unintentionally and for scientific study. Once inside the habitat, that dust will slowly settle depending on particle size and gravitational forces. Furthermore, dust is expected to be removed by air filtration, presumably by high-efficiency particulate air (HEPA) filters or their equivalent. As a result, exposures of no more than a few hours to dust that could be an ocular irritant are anticipated; however, those exposures could occur up to 5 days per week for 26 weeks or more as astronauts return from their work on the surface of a celestial body. To determine the potential hazard caused by these exposures, NASA decided to evaluate the potential for lunar dust to cause chemical and/or mechanical injury to the eye.

## Methods

### Test Material

**Figure 2:**
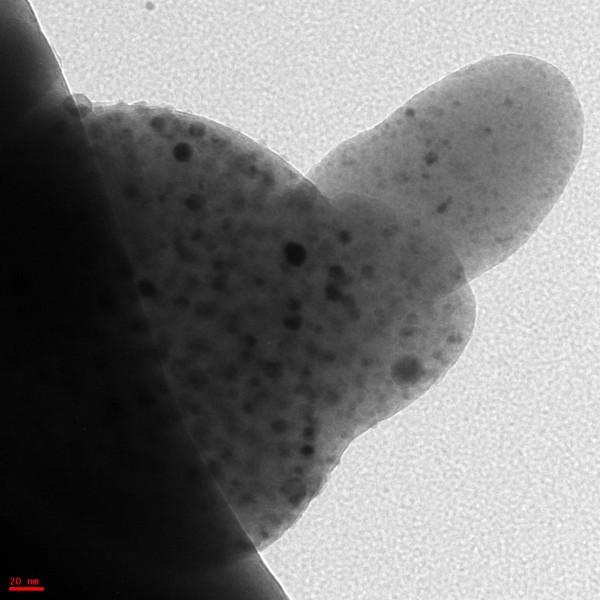
**Apollo 14 lunar dust image.** A scanning electron micrograph image of authentic lunar dust with iron metal inclusions.

The parent sample consisted of soil from the Apollo 14 mission (sample no. 14003,96). Figure 2 shows a scanning electron micrograph of the dust containing iron metal inclusions. The material was separated by pneumatic means within a glovebox containing ultrapure nitrogen (0.5 ppm H_2_O, 20.6 ppm O_2_), using the technique described in Cooper et al. [4]. To determine the mechanical irritancy or abrasiveness of the lunar dust, the fraction of material separated by pneumatic means described above with a mean particle size of 50.9 ± 19.8 μm was used for *in vivo* testing.

To prepare the sample for *in vitro* testing, an in-house jet-mill grinding method was used to grind coarse lunar soil grains to produce smaller particles and restore surface reactivity which may have been lost after years of storage. This method allowed us to test for the maximum chemical irritancy potential of the lunar dust, since grinding is expected to lead to a higher degree of surface reactivity due to the generation of silicon- or oxygen-based radicals (“dangling bonds”), which can react with water to produce hydroxyl radicals. It is also possible that grinding of lunar dust exposes reduced iron, which can react with oxygen and water to produce ROS, including hydrogen peroxide or superoxide [5]. The material was ground to a median particle diameter of 2.9 ± 1.0 μm. Previous work has shown that surface reactivity generated by grinding is greatly reduced or “passivated” over the course of a few hours by contact with humidity and atmospheric oxygen [5]. Consequently, all handling of the lunar soil (both separation and grinding) was performed in an ultrapure nitrogen environment to minimize its exposure to reactive atmospheric species until the tissues were dosed.

### *In vitro* testing

*In vitro* ocular testing was performed by Stillmeadow, Inc. using the commercially available EpiOcular^TM^ model. This model utilizes human-derived epidermal keratinocytes that have been cultured to form a stratified corneal epithelium [6]. Cell viability following exposure to lunar dust was determined by conversion of 3-[4,5-dimethylthiazol-2-yl]2,5-diphenyltetrazolium bromide (MTT) and was expressed as a percentage relative to untreated (negative control) tissues.

*Tissue Exposure* - MatTek assay medium (MTT-100-ASY) was pre-warmed to 37°C in a 5% CO_2_ incubator, and 0.9 mL was aliquoted into each well of a sterile 6-well plate. Each insert, containing room-temperature EpiOcular^TM^ tissue, was aseptically placed in one of six wells containing pre-warmed assay media and incubated for one hour at 37°C, under 5% CO_2_. Following incubation, the media was aspirated and then replaced with an identical volume of pre-warmed media. Approximately 100 mg of ground lunar dust, was applied in duplicate to EpiOcular™ tissues for each exposure time of 3, 30 and 60 minutes, as recommended by the manufacturer (MatTek). Additional tissues were dosed in duplicate each with exposure times of 3, 30 and 60 minutes to serve as dust controls using the following: sodium dodecyl sulfate (SDS) (Mfg: Fisher Bioreagents; Lot: 094466; Exp: Jan 2020), sodium hydroxide (NaOH) (Mfg: MP Biomedicals, LLC; Lot: 7367 J; Exp: Jan 2020) and hydrated amorphous silica (Mfg: Bel-Art Products; CAS 112926-00-8/7631-86-9; Exp: Jan 2020). Two tissues were exposed to 100 μL of deionized (DI) water for 60 minutes to serve as a negative control. The positive control, 0.3% Triton X-100, was used to dose two tissue replicates for each exposure time of 3, 30 and 60 minutes.

After exposure of the tissues was complete, tissues were gently rinsed with calcium- and magnesium-free phosphate buffered saline (PBS) until all test material was removed from each tissue insert, and any liquid remaining on the tissues was aspirated off. Each insert was then submerged in pre-warmed assay media and incubated for 10 minutes at 37°C, under 5% CO_2_.

Supplied MTT concentrate (MTT-100-CON) was thawed, diluted with the provided diluent (MTT-100-DIL), and mixed well. Three hundred microliters of MTT solution were added to each well of a sterile 24 well plate, and both it and a second empty 24 well plate, to be used in the extraction process, were labelled appropriately for each insert.

Following the 10 minute incubation, tissues were placed in a sterile 24-well plate containing 300 μl MTT solution per well, and returned to a 37°C, 5% CO_2_ incubator for 3 hours. Following the 3 hour MTT incubation, the tissues were gently rinsed with PBS to remove any remaining MTT solution and blotted with a Kimwipe. Tissue inserts were then added to the pre-labelled 24-well plate, immersed in 2 mL extractant solution (MTT-100-EXT) per well, and sealed inside a zip-top plastic bag to prevent evaporation. The tissues were allowed to extract overnight at room temperature in the dark.

*Direct MTT Reduction* - A decrease in MTT reduction capacity is used as the indicator of potential irritancy. Therefore, it is important to determine the MTT reduction potential of the test substance itself. To accomplish this, 100 mg of SDS, amorphous silica, NaOH and lunar dust were each added to Eppendorf tubes containing 1 mL MTT solution. One hundred μL of DI water were added to 1 mL MTT solution as a negative control. Tubes were placed in the dark at room temperature for approximately 60 minutes, and then assessed for color change to purple, indicative of auto-reduction. Any direct MTT reduction was measured photometrically and subtracted from the values obtained for the treated tissues.

*Determination of ET-50* - After transfer of tissue inserts to MTT plates and extraction were completed, any extraction solution remaining in the inserts was decanted back into the well and mixed thoroughly. Inserts were then discarded, and 200 μL from each well of each replicate were added to a 96 well plate in triplicate. Reduced MTT was then quantified photometrically. The optical density (OD) was measured at 570 nm using a dual wave length MRX Revelation spectrophotometer. The mean of the ODs for the two replicates for each substance and time point were used to calculate the ET-50 of all tissues using the manufacturer-provided spreadsheet. Results were expressed as percent viability in the lunar dust treated tissues relative to the negative control.

*Extrapolation of ET-50 to irritation potential* - A proprietary spreadsheet-based model was used to correlate the ET-50 value with the appropriate Draize irritation score. This model was developed by the manufacturer and based on their testing [7].

### *In vivo* testing

*In vivo* ocular testing was performed by Stillmeadow, Inc. according to U.S. Environmental Protection Agency guidelines (OPPTS 870.2400) [8]. All procedures were in compliance with Animal Welfare Act Regulations and were approved by the NASA and Stillmeadow, Inc. Institutional Animal Care and Use Committees (IACUC).

*Test Substance Administration* - Three healthy albino rabbits were released from quarantine five days after receipt. On Day 0, both eyes of each animal were carefully examined prior to treatment using a fluorescein sodium ophthalmic solution and cobalt-filtered light. Tetracaine HCl Ophthalmic Solution (0.5%, Bausch & Lomb, Lot 133541, Exp Sep 2012) was applied immediately prior to the fluorescein staining, with photographs of each eye taken both prior to and following fluorescein staining. Only those animals without extant eye defects or irritation were selected for testing. Each rabbit received 0.1 mg/kg Buprenorphine (Hospira, Lot 77531LL, Exp Sep 2012) parenterally as an analgesic at least 45 minutes prior to dosing.

As recommended by OECD guideline 405, Acute Eye Irritation/Corrosion, a dose equivalent to 0.1 mL volume, or 70 ± 2 mg of lunar dust, was placed into the conjunctival sac of the right eye of each animal by gently pulling the lower lid away from the eyeball to form a cup into which the test substance was poured [9]. The lids were gently held together for several seconds to minimize loss of material through the blinking reflex. The untreated left eyes served as comparative controls.

Observations – Gross observation of the treated eyes of all animals were examined without magnification under white room lighting provided by daylight-type fluorescent ceiling fixtures, and (if needed) an additional source of white light affixed to the examination table or using a handheld flashlight. All treated eyes were washed with room temperature DI water for one minute immediately after recording the 1-hour scores. If the test substance was still visible in the rabbit eye after the 1-minute wash, washing was continued for a maximum of 1 additional minute (2 minutes total). Photographs of each eye were taken at the 1-hour observation to document the presence or absence of residual test substance after washing and at each observation period through study termination.

The grades of ocular reaction were recorded at 1, 24, 48 and 72 hours after treatment. The corneas of all treated eyes were examined immediately after the 1- and 24-hour observations with a fluorescein sodium ophthalmic solution. A Finoff ocular transilluminator with cobalt blue filter (Welch Allyn, Skaneateles Falls, NY) was utilized to enhance visualization of fluorescein staining. Slit lamp exams of any fluorescein-stained abrasions were planned, but no abrasions were noted.

*Irritation Scoring Method* - Individual irritation scores for each animal at each scheduled observation were determined using the standard Draize scale of ocular irritancy (Appendix A). An average irritation score for each scheduled observation was then determined, with a maximum average irritation score derived from the observation yielding the highest average irritation score. The maximum average irritation score was used to rate the test substance according to the definitions presented in Appendix B.

## Results and Discussion

### Chemical Irritancy of Lunar Dust

OECD guideline 405 recommends that sequential testing, including the use of validated *in vitro* tests of corrosion/irritation, should be conducted prior to *in vivo* testing [9]. Therefore, the EpiOcular^TM^ eye irritation assay [6], was used to determine the relative chemical irritation produced by lunar dust prior to *in vivo* testing. This human-derived *in vitro* model has been used worldwide by chemical, pharmaceutical and consumer product companies in testing approaches to reduce or replace animal testing [10,11]. Results reported by Stillmeadow, Inc. are shown in Table 1. The ET-50 for lunar dust was greater than 60 minutes, and the dust was therefore classified as mildly irritating. In contrast, the ET-50 for known chemical irritants, such as NaOH and SDS, was less than 3 minutes.

**Table 1:** **Test results from EpiOcular *in vitro *testing**

	**Lunar Dust**	**Neg. Control**	**Pos. Control**	**NaOH**	**SDS**	**Amorphous silica**
3 min	1.117	N/A	0.931	0.014	0.275	1.465
30 min	1.023	N/A	0.548	0.011	0.029	1.288
60 min	1.013	1.077	0.187	0.010	0.012	1.449
ET-50	>60 min	N/A	30.6 min	<3 min	<3 min	>60 min
Irritancy	Minimal	N/A	Mild	Severe	Severe	Minimal

Average OD readings at 570 nm are presented for samples exposed to each test article for 3, 30, or 60 minutes. The ET-50 was estimated from the average OD reading according to the manufacturer’s spreadsheet. Chemical irritancy classification was then assigned according to the corresponding ET-50.

Although *in vitro* ocular toxicity testing provides useful information, it does have some limitations. The EpiOcular^TM^ assay evaluates only a single endpoint (cell viability) in corneal epithelial cells. Additional endpoints, such as hyperemia, swelling and mechanical injury, effects on other ocular structures, such as the conjunctiva, and physiological processes, such as blink response and tearing, can be assessed only by using intact eyes.

### Mechanical Irritancy of Lunar Dust

An acute eye irritation study was performed in New Zealand White albino rabbits to determine the mechanical irritancy of lunar dust in an intact eye and to confirm the results of the *in vitro* testing. The maximum average irritation score, using the Draize grading scale [12], was 4.0 at the 1-hour observation point for all three animals tested (Table 2). This score was due to slight redness and swelling of the conjunctiva seen only at the 1-hour observation time. No corneal scratching was observed by fluorescein staining. No adverse signs or symptoms were noted in the cornea, iris, or conjunctiva at any of the subsequent observation times (24, 48, and 72 hours). The lunar dust was rated as minimally irritating based on the maximum average irritation score, which is equivalent to “Not Classified (NC) for eye irritation” under the Globally Harmonized Classification system (UN-GHS) [13]. These results are consistent with the *in vitro* results and provide additional support that the EpiOcular assay system adequately predicts irritancy potential despite its limitations [14]. However, it is noted that as lunar grains become smaller, they become more rounded in shape and may lack abrasivity. This could explain the observed lack of irritancy.

**Table 2:** **Maximum average irritation scores from *in vivo *testing of lunar dust**

**Time After Treatment**	**Rabbit Number**			**Average Score**
	**0226-M**	**0230-M**	**0275-F**	
Hour 1	4	4	4	4.0
Hour 24	0	0	0	0.0
Hour 48	0	0	0	0.0
Hour 72	0	0	0	0.0
Maximum Average Irritation Score:			4.0	Minimally irritating

Average irritation scores are presented for each of the three animals at each observation time. Minor conjunctival redness and swelling were noted in all animals 1 hour post-exposure. According to the grading scale in Appendix A, this corresponds to an irritation score of 4. All redness and swelling resolved within 24 hours.

M – Male; F – Female.

These results are consistent with those for similar terrestrial dusts, such as volcanic ash, which is often used as a simulant for lunar dust. After the eruption of Mount St. Helens in 1980, ophthalmologists observed that ash particles were well-tolerated by those exposed, producing acute irritation but no long-term ocular effects [15]. Similarly, a recent 10-year survey of ocular effects in school-aged children exposed to volcanic ash reported that those in high-exposure areas did have a higher incidence of ocular symptoms, but these were limited to minor, acute effects (redness, itching, foreign-body sensation, and discharge) that were readily treatable with eye drops [16]. Desert sands are similarly acutely irritating to the eye [17].

There are limitations to our study. Only one source of lunar dust, generally representative of low-Titanium mare lunar dust, was tested. Dust from the highlands area of the lunar surface has a substantially different mineral content, and therefore, these results may not be representative of that dust nor of dust from exotic locations such as the permanently dark areas in the basins of craters near the poles.

## Conclusions

These results suggest that lunar dust like that returned aboard Apollo 14 will not cause significant eye irritation once nominal operations commence on the lunar surface. No special precautions are recommended to protect against ocular exposures; however, it is noted that contact lens wearers may be more susceptible to irritation from dust that may become trapped under the lens. Fully shielded goggles may be used if dust becomes a nuisance. If irritating grains do enter the eye, an eyewash station is recommended to remove the offending dust. It is important to note that this study focused exclusively on the ocular toxicity of lunar dust. Other potential routes of exposure, including inhalation and dermal irritation, are being examined.

**Table 3:** The total score for the eye is the sum of all scores obtained for the cornea, iris, and conjunctivae, with the possible maximum total score for the eye being equal to 110

I. Cornea (Maximum score = 80; A x B x 5)	
**A. Opacity‐degree (area most dense taken for reading)**	
No opacity	0
Slight dulling of normal luster	+
Scattered or diffuse areas of opacity (other than slight dulling of normal luster), details of iris clearly visible	1
Easily discernible translucent area, details of iris slightly obscured	2
Nacreous area, no details of iris visible, size of pupil barely discernible	3
Opaque cornea, iris not discernible through the opacity	4
**B. Area of cornea involved**	
One quarter (or less), but not zero	1
Greater than one quarter, but less than half	2
Greater than half, but less than three quarters	3
Greater than three quarters, up to whole area	4
**C. Fluorescein staining‐appearance of yellow-green staining of cornea**	
Cornea not examined with fluorescein	-
No fluorescein staining	0
Positive fluorescein staining	P
Area of cornea involved	
One quarter (or less), but not zero	A
Greater than one quarter, but less than half	B
Greater than half, but less than three quarters	C
Greater than three quarters, up to whole area	D
**D. Stippling‐appearance of pinpoint roughening**	
No stippling	0
Presence of stippling	S
Area of cornea involved	
One quarter (or less), but not zero	A
Greater than one quarter, but less than half	B
Greater than half, but less than three quarters	C
Greater than three quarters, up to whole area	D
II. Iris (Maximum score = 10; A x 5)	
**A. Grades**	
Normal	0
Markedly deepened rugae, congestion, swelling, moderate circumcorneal hyperemia or injection (any of these or combination thereof), iris still reacting to light (sluggish reaction is positive)	1
No reaction to light, hemorrhage, gross destruction (any or all of these)	2
III. Conjunctivae (Maximum score = 20; (A+B+C) x 2)	
**A. Redness (refers to palpebral and bulbar conjunctivae excluding cornea and iris)**	
Blood vessels normal	0
Some blood vessels definitely hyperemic (injected)	1
Diffuse, crimson color, individual vessels not easily discernible	2
Diffuse beefy red	3
**B. Chemosis: lids and/or nictitating membrane**	
No swelling	0
Any swelling above normal (includes nictitating membrane)	1
Obvious swelling with partial eversion of lids	2
Swelling with lids about half closed	3
Swelling with lids more than half closed	4
**C. Discharge**	
No discharge	0
Any amount different from normal (does not include small amounts observed in inner canthus of normal animals)	1
Discharge with moistening of the lids and hairs just adjacent to lids	2
Discharge with moistening of the lids and hairs, and considerable area around the eye	3
**D. Necrosis or ulceration of the palpebral and bulbar conjunctivae or nictitating membrane**	
No necrosis or ulceration	0
Presence of necrosis or ulceration	N

## Appendices

### Appendix A-Grading Scale for the Acute Eye Irritation Study in Rabbits^12^

### Appendix B-Rating of Test Substance Based on Eye Irritation

Non-irritating (0.0‐0.5) - To maintain this rating, all scores at the 24-hour reading must be zero; otherwise, increase rating one level.

Practically non-irritating (>0.5‐2.5) - To maintain this rating, all scores at the 24-hour reading must be zero; otherwise, increase rating one level.

Minimally irritating (>2.5‐15.0) - To maintain this rating, all scores at the 72-hour reading must be zero; otherwise, increase rating one level.

Mildly irritating (>15.0‐25.0) - To maintain this rating, all scores at the 7-day reading must be zero; otherwise, increase rating one level.

Moderately irritating (>25.0‐50.0) - To maintain this rating, scores at the irritating 7-day reading must be less than or equal to 10 for 60% or more of the animals; also, the 7-day mean score must be less than or equal to 20. If the 7-day mean score is less than or equal to 20, but less than 60% of the animals show scores less than or equal to 10, then no animal with a score greater than 10 can exceed a score of 30 if rating is to be maintained; otherwise, increase rating one level.

Severely irritating (>50.0‐80.0) - To maintain this rating, scores at the irritating 7-day reading must be less than or equal to 30 for 60% or more of the animals; also, the 7-day mean score must be less than or equal to 40. If the 7-day mean score is less than or equal to 40, but less than 60% of the animals show scores less than or equal to 30, then no animal with a score greater than 30 can exceed a score of 60 if rating is to be maintained; otherwise, increase rating one level.

Extremely irritating (>80.0‐110.0)

NOTE: The rating of the test substance is not to be increased more than one level above its maximum average score.

### Competing interests

The authors declare that they have no competing interests.

## Authors' contributions

VM, HG, KM, and JJ participated in the design of the study. BC prepared appropriate lunar samples for testing. KM served as the study coordinator for Stillmeadow, Inc., where the EpiOcular^TM^ assay and the *in vivo* ocular testing were carried out. VM and HG coordinated the study and manuscript development. All authors contributed text and read and approved the final manuscript.

## Authors' information

JJ, HG, and VM are certified in general toxicology by the American Board of Toxicology. JJ completed a Ph.D. at the University of Maryland in 1981 and has served as NASA’s Chief Toxicologist for 22 years. HG completed a Ph.D. at the University of Texas Graduate School of Biomedical Sciences, Houston, in 1979 and has worked in the Space Toxicology Office at the Johnson Space Center for 21 years. VM completed a Ph.D. at the University of Alabama at Birmingham in 2004 and has worked in the Space Toxicology Office for 3 years. BC completed a Ph.D. at the University of Texas at Dallas in 1992 and is a lunar soil scientist at NASA’s Johnson Space Center. KM completed a MS at LSU Health Sciences Center, New Orleans in 2006 and since 2008 has served as the Director of Microbiology at Stillmeadow, Inc.

## Pre-publication history

The pre-publication history for this paper can be accessed here:

http://www.biomedcentral.com/1471-2415/12/26/prepub
